# Streptozotocin-induced Diabetes Represses Hepatic CYP2R1 Expression but Induces Vitamin D 25-Hydroxylation in Male Mice

**DOI:** 10.1210/endocr/bqac060

**Published:** 2022-05-07

**Authors:** Mahmoud-Sobhy Elkhwanky, Outi Kummu, Jukka Hakkola

**Affiliations:** Research Unit of Biomedicine, Pharmacology and Toxicology, University of Oulu, Oulu, Finland; Medical Research Center Oulu, Oulu University Hospital and University of Oulu, Oulu, Finland; Biocenter Oulu, University of Oulu, Oulu, Finland; Research Unit of Biomedicine, Pharmacology and Toxicology, University of Oulu, Oulu, Finland; Medical Research Center Oulu, Oulu University Hospital and University of Oulu, Oulu, Finland; Biocenter Oulu, University of Oulu, Oulu, Finland; Research Unit of Biomedicine, Pharmacology and Toxicology, University of Oulu, Oulu, Finland; Medical Research Center Oulu, Oulu University Hospital and University of Oulu, Oulu, Finland; Biocenter Oulu, University of Oulu, Oulu, Finland

**Keywords:** vitamin D metabolism, diabetes, streptozotocin, CYP2R1, 25-hydroxyvitamin D

## Abstract

Vitamin D deficiency [ie, low plasma 25-hydroxyvitamin D (25-OH-D)] associates with the prevalence of metabolic diseases including type 1 diabetes; however, the molecular mechanisms are incompletely understood. Recent studies have indicated that both fasting and metabolic diseases suppress the cytochrome P450 (CYP) 2R1, the major hepatic vitamin D 25-hydroxylase. We specifically studied the effect of a mouse model of type 1 diabetes on the regulation of *Cyp2r1* and vitamin D status. We show that streptozotocin-induced diabetes in mice suppresses the expression of the *Cyp2r1* in the liver. While insulin therapy normalized the blood glucose levels in the diabetic mice, it did not rescue the diabetes-induced suppression of *Cyp2r1*. Similar regulation of *Cyp2r1* was observed also in the kidney. Plasma 25-OH-D level was not decreased and was, in contrast, higher after 4 and 8 weeks of diabetes. Furthermore, the vitamin D 25-hydroxylase activity was increased in the livers of the diabetic mice, suggesting compensation of the *Cyp2r1* repression by other vitamin D 25-hydroxylase enzymes. *Cyp27b1*, the vitamin D 1α-hydroxylase, expression in the kidney and the plasma 1α,25-dihydroxyvitamin D level were higher after 4 weeks of diabetes, while both were normalized after 13 weeks. In summary, these results indicate that in the mouse model of type 1 diabetes suppression of hepatic *Cyp2r1* expression does not result in reduced hepatic vitamin D 25-hydroxylase activity and vitamin D deficiency. This may be due to induction of other vitamin D 25-hydroxylase enzymes in response to diabetes.

Vitamin D is a prohormone produced in the skin in response to the sunlight UV-B or, alternatively, received through diet and subsequently activated in 2 enzymatic steps (1). The activation of vitamin D is initiated with 25-hydroxylation in the liver, catalyzed mainly by cytochrome P450 (CYP) 2R1, and followed by renal 1α-hydroxylation, catalyzed by CYP27B1, to yield the main active form 1α,25-dihydroxyvitamin D [1α,25(OH)_2_D] 1).

CYP2R1 is considered as the major vitamin D 25-hydroxylase in the liver (2). In humans, genetic mutation in the *CYP2R1* gene causes an inherited form of vitamin D deficiency and rickets in children (3, 4), and genome-wide association studies have identified *CYP2R1* gene variants as 1 of the key genetic determinants of low 25-hydroxyvitamin D (25-OH-D) levels (5, 6). In mice, *Cyp2r1* knockout reduced the plasma 25-OH-D by 50%, which indicates that the CYP2R1 enzyme is a major, but not the exclusive, vitamin D 25-hydroxylase, and other CYPs beside CYP2R1 could 25-hydroxylate vitamin D (2).

Vitamin D deficiency is considered as one of the most prevalent medical conditions worldwide (7). Vitamin D status is usually assessed by measuring the main circulating vitamin D metabolite 25-OH-D because of its long half-life of about 2 weeks (8). CYP2R1 has been traditionally considered to be constitutively expressed, and thus 25-OH-D level would reflect the global supply of vitamin D (9). However, recent studies, including ours, have challenged this concept and demonstrated that CYP2R1 expression and vitamin D 25-hydroxylation are regulated by energy homeostasis (10-12). At the molecular level, peroxisome proliferator-activated receptor gamma coactivator 1-α (PGC-1α)/estrogen-related receptor α (ERRα) pathway and glucocorticoid receptor (GR) activation were identified as *Cyp2r1* repressive mechanisms (10).

We previously demonstrated that CYP2R1 expression was suppressed in the mouse liver by fasting, high-fat diet (HFD)-induced obesity and streptozotocin (STZ)-induced diabetes (10). Furthermore, in the obese mice, the plasma 25-OH-D level was reduced, and in the livers of fasting mice, the vitamin D 25-hydroxylase activity was strongly reduced (10, 11) Moreover, Roizen et al reported that livers of the HFD-fed, obese mice had reduced vitamin D 25-hydroxylase activity (12). These data thus support the notion that downregulation of CYP2R1 expression associates with reduced vitamin D 25-hydroxylase activity and could at least partially explain why the vitamin D deficiency is commonly associated with obesity and diabetes.

As mentioned earlier, we have reported that STZ-induced, insulin-deficiency diabetes represses hepatic CYP2R1 expression, but the effect on vitamin D metabolism was not studied further (10). The current study was designed, first, to study effect of STZ-induced diabetes in mice on *Cyp2r1* expression, vitamin D status, and hepatic vitamin D 25-hydroxylase activity. Second, we aimed to study whether insulin therapy could rescue the effect of STZ-induced diabetes on *Cyp2r1* expression. Since we originally expected that the suppression of *Cyp2r1* would result in reduced hepatic vitamin D 25-hydroxylase activity, we also tested whether high-dose vitamin D substitution could overcome potential deficiencies in the vitamin D 25-hydroxylation.

## Materials and Methods

### Animal Experiments

C57BL/6N male mice were housed in individual cages in standard conditions with 12-hour dark:light cycle. Mice were obtained from the Laboratory Animal Center, University of Oulu. At the end of the experiments, mice were sacrificed by CO_2_ inhalation and neck dislocation. The blood was drawn into EDTA-primed syringe from vena cava and tissues collected and snap-frozen in liquid nitrogen. All animal procedures have been approved by the Animal Experiment Board, Finland (license no. ESAVI/8240/04.10.07/2017).

### Streptozotocin Treatment

Male mice, aged 8 to 10 weeks, were injected with vehicle (50 mM sodium citrate buffer pH 4.5) or 60 mg/kg STZ (Sigma-Aldrich, St. Louis, MO, USA) intraperitoneally once daily for 5 successive days. To reduce the weight loss and improve survival, the mice were kept in a warm room (temperature 29°C) and were given hard and soft chow diet (the chow food was softened with water). The mice were closely monitored by measuring the blood glucose and weight. The mice were followed for 4 (vehicle n = 10, STZ n = 13), 8 (vehicle n = 10, STZ n = 20), and 13 (vehicle n = 10, STZ n = 34) weeks counted from the first STZ injection. During the 13-week experiment, for the last 4 weeks before sacrificing, the diabetic mice were divided into 3 groups: (1) untreated (STZ = 14), (2) treated with long-acting insulin (Ins) injection (Lantus® insulin glargine) 100 units/mL, Sanofi) 1 IU subcutaneously twice daily (STZ + Ins n = 10), and (3) treated with oral vitamin D3 (VD) [JekoVitD3 2440 IU/mL, vitamin D3 (Orion Corporation, Finland) fortified with extra vitamin D3 (Sigma) to yield the final concentration 20000 IU/mL] 500 IU 3 times/week (STZ + VD n = 10). The control and the STZ groups are unequal because a few extra mice were included to the STZ groups to prepare for possible loss of mice. However, no animals were lost during the experiment. Blood glucose measurements were done from blood samples taken from the vena saphena using FreeStyle Precision Neo glucometer and FreeStyle Precision glucose strips (Abbott Laboratories). Urine samples were collected during the sacrifice, and glucose and ketone bodies were measured with Mission® Urinanalysis Reagent strips (Acon Laboratories) according to the manufacturer’s instructions.

### RNA Preparation and Quantitative Reverse Transcription-Polymerase Chain Reaction

Total RNA was extracted from tissues using RNAzol RT reagent according to the manufacturer’s protocol (Sigma-Aldrich, St. Louis, MO, USA). One microgram of RNA was used for complimentary DNA synthesis with p(dN)6 random primers (Roche Diagnostics, Mannheim, Germany) using RevertAid complimentary DNA synthesis kit according to the manufacturer’s protocol (Thermo Fisher Scientific, Waltham, MA, USA). Quantitative real time-polymerase chain reactions were conducted using SYBR Green chemistry or TaqMan chemistry (Applied Biosystems, Foster City, CA, USA). A 2-step amplification program was conducted with the following steps: 1 second at 95°C and 20 seconds at 60°C for 40 cycles (SYBR assay) or 15 seconds at 95°C and 1 minute at 60°C for 40 cycles (TaqMan assay). Predenaturation step for 10 minutes at 95°C was conducted before the amplification program started. Sequences for the primers and the TaqMan assays are listed in Table 1. The fluorescence values of the quantitative polymerase chain reaction products were corrected with the fluorescence signals of the passive reference dye. The messenger RNA (mRNA) levels of target genes were normalized against *Tbp* (TATA-box binding protein), *18S*, or *Gapdh* (glyceraldehyde-3-phosphate dehydrogenase) reference genes levels using the comparative C_T_ (ΔΔC_T_) method.

**Table 1.** Sequences of the quantitative polymerase chain reaction primers

Gene	Forward primer (5’to 3’)	Reverse primer (5’to 3’)
*Cyp2r1*	Mm01159414_m1(Life Technologies)	
*Cyp24a1*	CTGCCCCATTGACAAAAGGC	CTCACCGTCGGTCATCAGC
*Cyp27b1*	TCCTGGCTGAACTCTTCTGC	GGCAACGTAAACTGTGCGAA
*Gapdh*	GGTCATCATCTCCGCCCC	TTCTCGTGGTTCACACCCATC
*Gr*	AGCTCCCCCTGGTAGAGAC	GGTGAAGACGCAGAAACCTTG
*Pepck*	GGTGTTTACTGGGAAGGCATC	CAATAATGGGGCACTGGCTG
*Pgc-1α*	TCCTCCTCATAAAGCCAACC	GCCTTGGGTACCAGAACACT
*18S*	CGCCGCTAGAGGTGAAATTC	CCAGTCGGCATCGTTTATGG
*Tat*	TGCTGGATGTTCGCGTCAATA	CGGCTTCACCTTCATGTTGTC
*Tbp*	GAATATAATCCCAAGCGATTTG	CACACCATTTTTCCAGAACTG
*Vdbp*	CCTGCTGGCCTTAGCCTTT	TGCTCAAATGTGCTACTGGAAA
*Vdr*	GAATGTGCCTCGGATCTGTGG	GGTCATAGCGTTGAAGTGGAA

### Measurement of the Plasma Vitamin D Metabolites

The plasma levels of 25-OH-D and 1α,25(OH)_2_D levels were measured using Vitamin D^s^ enzyme immunoassay kit (RRID:AB_2756867, https://scicrunch.org/resources/data/record/nif-0000-07730-1/AB_2756867/resolver?q=AB_2756867&l=AB_2756867&i=rrid:ab_2756867-2692323 and RRID:AB_2891249, https://scicrunch.org/resources/data/record/nif-0000-07730-1/AB_2891249/resolver?q=AB_2891249&l=AB_2891249&i=rrid:ab_2891249-2826705, respec tively; Immunodiagnostic System, Tyne & Wear, UK) according to the manufacturer’s protocol. 25-OH-D measurements were conducted in duplicates, while 1α,25(OH)_2_D measurements were conducted in a single replicate because of the limited amount of plasma.

### Vitamin D 25-Hydroxylase Assay

Microsomes were extracted using differential centrifugation (13). The microsomal protein concentration was measured by Bradford assay (Bio-Rad, Hercules, CA, USA). Mouse liver microsomal protein samples (0.5 mg/mL) were subjected to incubation with 0.5 µmol/L cholecalciferol (vitamin D3) together with 0.1 mol/L phosphate-buffer and preincubated for 5 minutes. The enzymatic reactions were started by adding 0.5 mmol/L nicotinamide adenine dinucleotide phosphate. The reactions were incubated for 40 minutes at 37°C with shaking. To stop the reaction, ice-cold acetonitrile at a 1:1 volume ratio was added, mixed well, and stored in liquid nitrogen until analysis. Quantitative analysis of the resultant 25-OH-D was conducted using liquid chromatography-tandem mass spectrometry as described previously (10).

### Histology

Liver paraffin sections (5 µm) were subjected to hematoxylin and eosin staining with Mayer’s hematoxylin. Paraffin-embedded mouse liver cross-section were used for immunostaining with anti–vitamin D receptor (VDR) antibody (Abcam cat no. ab3508, RRID:AB_303857, https://scicrunch.org/resources/data/record/nif-0000-07730-1/AB_303857/resolver?q=AB_303857&l=AB_303857&i=rrid:ab_303857-1984234) 1:1000 dilution for 30 minutes at room temperature. En Vision peroxidase/diaminobenzidine detection system (Dako cat no. K5007) with chromogenic substrate diaminobenzidine was used for visualization. Before antibody incubation, antigen retrieval was performed with 10 mM sodium citrate buffer pH 6 with heating near boiling point for 10 minutes, and endogenous peroxidase activity was quenched with peroxidase blocking solution (Dako cat no. S2023) for 5 minutes. The sections were counterstained with hematoxylin.

### Statistical Analysis

The statistical analysis was conducted using GraphPad Prism Software (La Jolla, CA, USA). The normal distribution of the data was tested using D’Agostino & Pearson test, and parametric tests were used only if the data were normally distributed. Student’s 2-tailed *t*-test was used to compare means of 2 groups except in Figures 8A and 8E and 9A and 9B, where the Mann-Whitney U test was used. One-way analysis of variance, followed Tukey’s post hoc test, was used to compare multiple groups, except in Figures 2E, 4C and 4D, 8B and 8F, and 9D, where Kruskal-Wallis test, followed by Dunn’s post hoc test, was used. Differences were considered significant at *P* < 0.05.

## Results

### Streptozotocin-induced Model of Type 1 Diabetes in Mice

**Figure 1.**
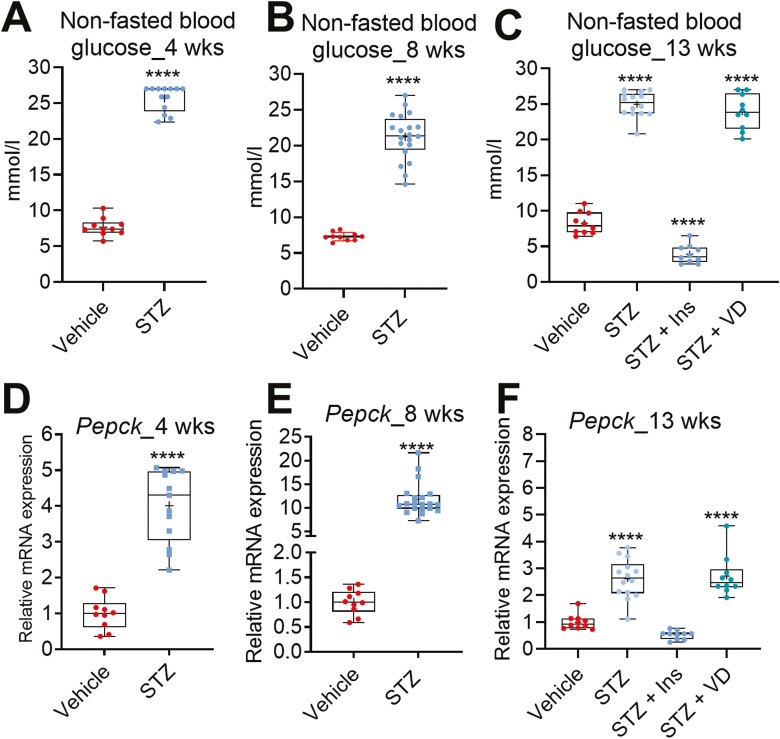
Streptozotocin treatment induces diabetes in mice. (A-C) Nonfasted blood glucose 4, 8, and 13 weeks after STZ treatment. (D-F) *Pepck* messenger RNA expression in the liver. The box-and-whisker plots indicate the minimum, 25th percentile, median,75th percentile, and maximum. In addition, the mean is indicated with +. *P < 0.05, **P < 0.01, ***P < 0.001, and ****P < 0.0001. Abbreviations: Ins, insulin; STZ, streptozotocin; VD, vitamin D3.

We studied the effect of STZ-induced diabetes model (14) on vitamin D metabolism in mice. Mice were sacrificed 4, 8, or 13 weeks after the first STZ injection. The STZ-injected mice started to display elevated blood glucose 1 week after the first injection (data not shown). In the 13-week experiment, a subset of the STZ mice was treated either with insulin injection twice daily or with oral vitamin D dose 3× per week for the last 4 weeks of the experiment. Before sacrificing the mice, the nonfasted blood glucose was measured from all mice. As a sign of diabetic phenotype, the STZ-injected mice (STZ mice) displayed very high glucose levels compared to the vehicle controls (nondiabetic mice) (Fig 1A-1C). As expected, insulin treatment normalized the blood glucose, while vitamin D had no effect (Fig 1C). Additionally, glucose was detected in the urine of all the STZ mice, while no glucose was detected in the nondiabetic controls (Tables 2 and 3). Ketone bodies were detected in the urine of some but not all the STZ-mice (Tables 2 and 3). Insulin treatment mostly eliminated the glucose and ketone bodies from the urine (Table 3).

**Table 2.** Glucose and ketone bodies in urine of the control and streptozotocin mice of the 4- and 8-week experiments

Mice, n	4 weeks		8 weeks	
	Glucose	Ketones	Glucose	Ketones
Nondiabetic control mice				
10	All neg	All neg	All neg	All neg
STZ				
1	+++	−	+++	+++
2	+++	−	+++	−
3	+++	−	++++	±
4	++++	−	++++	−
5	+++	−	++++	±
6	+++	−	++++	−
7	+++	−	++++	+
8	++++	++	+++	±
9	++++	+++	++++	−
10	+++	+	+++	−
11	++++	+	++++	+++
12	++	−	+++	−
13	++	+++	++++	++
14			++++	++++
15			++++	−
16			++++	+
17			++++	++
18			++	++++
19			+++	++++
20			+++	−

Glucose: − not detected, ± 5 mmol/L, + 15 mmol/L, ++ 30 mmol/L, +++ 60 mmol/L, and ++++ ≥110 mmol/L. Ketone bodies: − not detected, ±0.5 mmol/L, + 1.5 mmol/L, ++ 4.0 mmol/L, +++ 8.0 mmol/L, and ++++ 16 mmol/L.

Abbreviations: neg, negative; STZ, streptozotocin.

**Table 3.** Glucose and ketone bodies in urine of the control and streptozotocin mice of the 13-week experiment

Mice, n	13 weeks	
	Glucose	Ketones
Nondiabetic control mice		
10	All neg	All neg
STZ		
1	+++	−
2	++++	−
3	++++	−
4	++++	−
5	++++	−
6	++++	−
7	+++	−
8	+++	−
9	+++	−
10	++++	−
11	+++	−
12	++	+
13	++	++
14	+++	−
STZ + Ins		
1	±	−
2	+	±
3	−	−
4	+	−
5	±	−
6	±	−
7	−	±
8	−	−
9	+	−
10	−	−
STZ + VD		
1	++++	−
2	++	−
3	+++	−
4	++	−
5	++++	−
6	+++	−
7	+++	±
8	+++	±
9	+++	−
10	+++	±

Glucose: − not detected, ± 5 mmol/L, + 15 mmol/L, ++ 30 mmol/L, +++ 60 mmol/L, and ++++ ≥110 mmol/L. Ketone bodies: − not detected, ±0.5 mmol/L, + 1.5 mmol/L, ++ 4.0 mmol/L, +++ 8.0 mmol/L, and ++++ 16 mmol/L.

Abbreviations: Ins, insulin; neg, negative; STZ, streptozotocin; VD, vitamin D3.

Consistent with the increase in the blood glucose, the mRNA level of a key gluconeogenic gene phosphoenolpyruvate carboxykinase (*Pepck*) was elevated in the livers of the STZ mice (Fig 1D-1F), and the effect was abolished by the insulin treatment (Fig 1F). Altogether, these results indicate that the STZ treatment successfully induced a model of type 1 diabetes in the treated mice and that the insulin treatment improved the glucose homeostasis in the diabetic mice expectedly.

### STZ-induced Diabetes Represses C*yp2r1* Expression in the Liver

**Figure 2.**
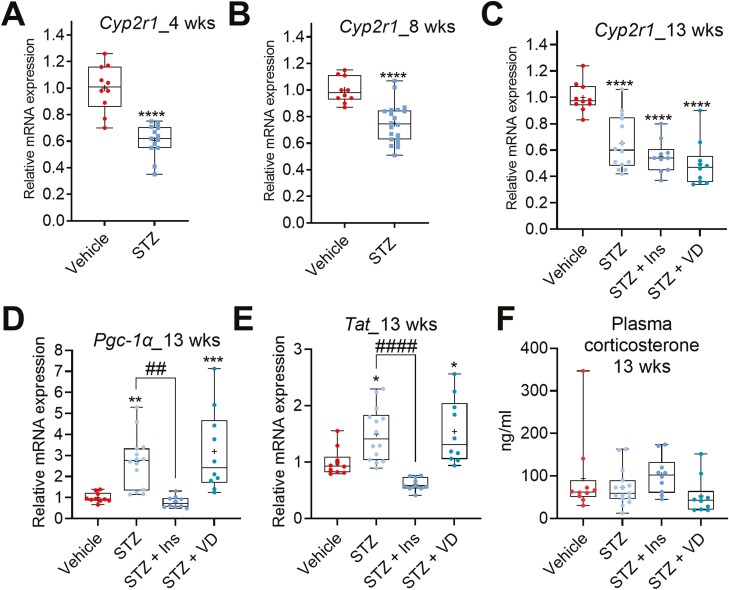
STZ-induced diabetes in mice represses *Cyp2r1* in the liver. (A-C) STZ-induced diabetes represses the *Cyp2r1* expression after 4, 8, and 13 weeks of STZ treatment. (D-F) Effect of STZ-induced diabetes on the liver expression of *Pgc-1α* and *Tat* messenger RNAs and the plasma corticosteroids levels in the 13-week experiment. The box-and-whisker plots indicate the minimum, 25th percentile, median, 75th percentile, and maximum. In addition, the mean is indicated with +. *P or #*P* < 0.05, **P or ##*P* < 0.01, ***P or ###*P* < 0.001, and ****P or ####*P* < 0.0001. Abbreviations: Ins, insulin; STZ, streptozotocin; VD, vitamin D3.

To analyze the effect of STZ-induced diabetes on vitamin D bioactivation in the liver, we measured the mRNA level of *Cyp2r1*, the major hepatic vitamin D 25-hydroxylase. The STZ-induced diabetes significantly repressed *Cyp2r1* expression in the liver by 39%, 25%, and 35% after 4, 8, and 13 weeks, respectively, compared to the nondiabetic mice (Fig 2A-C). Although treatment with insulin corrected the glucose level (Fig 1C), it did not rescue the repression of *Cyp2r1* in the livers of the STZ mice (Fig 2C). Furthermore, vitamin D treatment did not affect the *Cyp2r1* expression in the STZ mice (Fig 2C).

In our earlier studies, both the PGC-1α/ERRα pathway and GR activation were identified as *Cyp2r1* repressive mechanisms (10). Thus, we next measured expression of *Pgc-1α* and a GR target gene tyrosine aminotransferase (*Tat*) to analyze activation status of these regulatory pathways in response to the STZ-induced diabetes and the potential effect of insulin therapy. As expected, *Pgc-1α* mRNA was induced in the livers of the STZ mice, and the expression was normalized by the insulin therapy (Fig 2D). The GR target gene *Tat* mRNA was induced in the livers of the STZ-mice, and it was significantly repressed by insulin (Fig 2F). We also measured corticosterone level from the plasma collected at the time of sacrifice; however, none of the treatments had any effect. These results do not support the idea that either PGC-1α or GR play any major role in the repression of *Cyp2r1* expression in the STZ-induced diabetes.

### *Cyp2r1* Expression in the Liver Decreases With Age

**Figure 3.**
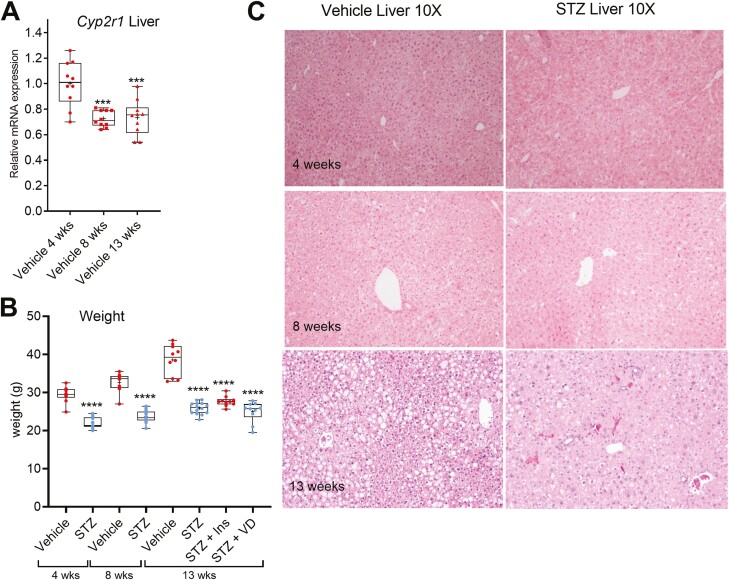
The basal *Cyp2r1* expression in the mouse liver decreases with age. (A) The *Cyp2r1* messenger RNA levels in the livers of nondiabetic (vehicle treated) mice of different age. The weeks indicate time point after vehicle treatment. (B) Weight of the mice 4, 8, and 13 weeks after streptozotocin treatment. (C) The hematoxylin and eosin staining of liver sections from the 4-, 8- and 13-week experiments, 10-fold magnification. The box-and-whisker plots indicate the minimum, 25th percentile, median, 75th percentile, and maximum. In addition, the mean is indicated with +. *P < 0.05, **P < 0.01, ***P < 0.001, and ****P < 0.0001. Abbreviations: Ins, insulin; STZ, streptozotocin; VD, vitamin D3.

The hepatic CYP2R1 abundance has been reported to be decreased with age (15); however, the mechanism is not yet clear. Therefore, we compared the relative basal *Cyp2r1* mRNA level in mice of different ages (control groups of the 4-, 8-, and 13-week experiments). Relative *Cyp2r1* mRNA was reduced significantly in the livers of nondiabetic mice after 8 and 13 weeks compared to the nondiabetic mice after 4 weeks (Fig 3A). Interestingly, by the end of the 13-week experiments, we noticed that the nondiabetic control mice started to look obese. The average weight of nondiabetic mice was 38.52 ± 4.1 g, compared to 25.9 ± 1.6 g in the STZ group (Fig 3B). Previous results have shown that HFD-induced obesity significantly represses *Cyp2r1* in the mouse liver (10-12). Noteworthy, although the basal expression level of *Cyp2r1* reduced in the older mice, the relative effect of the STZ-induced diabetes remained very similar (Fig 2C). Interestingly, hematoxylin and eosin staining of the liver sections indicated clear steatosis in the livers of control mice after the 13-week experiment (Fig 3C). Altogether, these data suggest that the basal *Cyp2r1* mRNA expression in the liver decreases with age, and it may be attributed to the development of obesity and lipid accumulation in the liver.

### STZ-induced Diabetes Increases Plasma 25-OH-D Level and Hepatic Vitamin D 25-Hydroxylase Activity

**Figure 4.**
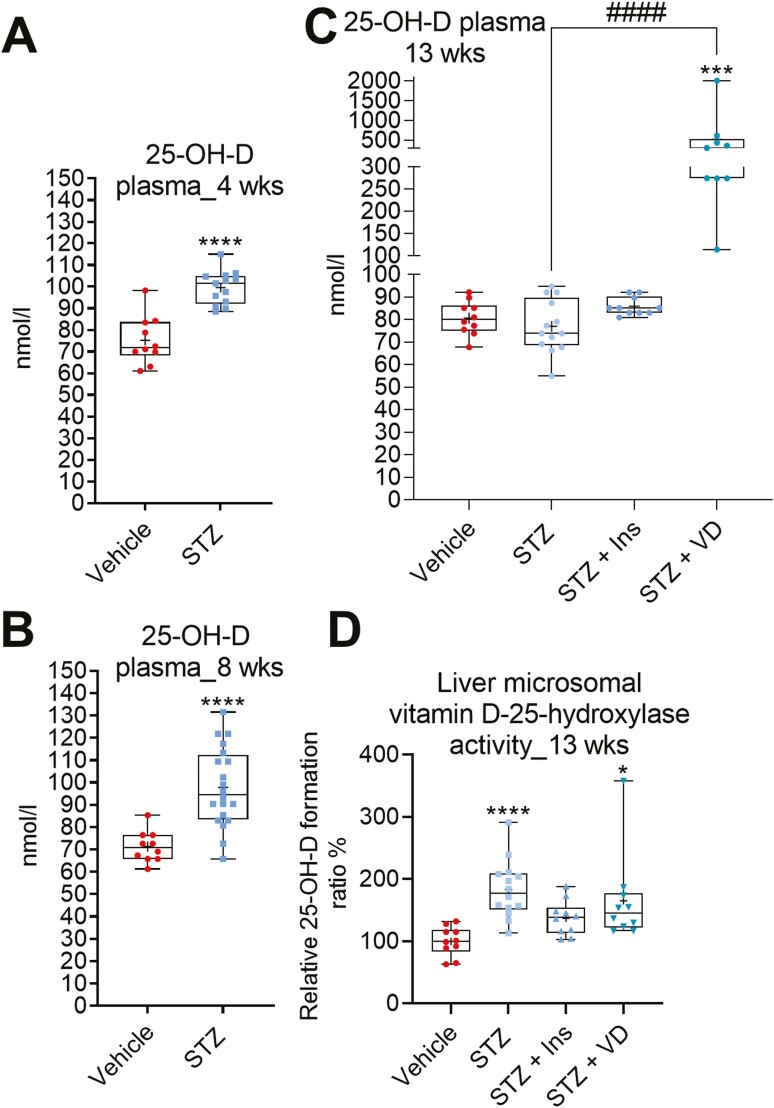
Effect of STZ-induced diabetes in mice on plasma 25-hydroxyvitamin D (25-OH-D). Plasma 25-OH-D levels after (A) 4 weeks, (B) 8 weeks, and (C) 13 weeks after STZ treatment. (D) The liver microsomal vitamin D 25-hydroxylase activity in the 13-week experiment. The box-and-whisker plots indicate the minimum, 25th percentile, median, 75th percentile, and maximum. In addition, the mean is indicated with +. *P or #*P* < 0.05, **P or ##*P* < 0.01, ***P or ###*P* < 0.001, and ****P or ####*P* < 0.0001. Abbreviations: Ins, insulin; STZ, streptozotocin; VD, vitamin D3.

Plasma 25-OH-D was measured to study the functional effect of *Cyp2r1* repression in the STZ mice. Unexpectedly, the plasma 25-OH-D was significantly higher in the STZ mice compared to nondiabetic mice after 4 and 8 weeks (Fig 4A and 4B), while the plasma 25-OH-D level did not differ after 13 weeks between the diabetic and nondiabetic animals (Fig 4C). Insulin had no effect on the plasma 25-OH-D levels in the STZ mice (Fig 4C). In contrast, vitamin D supplementation strongly increased the plasma 25-OH-D in the STZ mice (Fig 4C).

To investigate further the effect of STZ-induced diabetes on the vitamin D 25-hydroxylation step, we measured the liver microsomal vitamin D 25-hydroxylase activity in the mice of the 13-week experiment. In contrast to the liver *Cyp2r1* mRNA results, there was a significantly higher vitamin D 25-hydroxylase activity in the liver microsomes extracted from the STZ mice (Fig 4D). The effect of STZ-induced diabetes on vitamin D 25-hydroxylase activity was partially prevented by insulin treatment.

While CYP2R1 is the major vitamin D 25-hydroxylase (2), there are several other CYP enzymes considered as potential vitamin D 25-hydroxylases (ie, CYP27A1 and CYP3A4) (16). Thus, we analyzed the mRNA levels of *Cyp3a11* (the mouse orthologue of the human *CYP3A4*) and *Cyp27a1* in the livers of nondiabetic and diabetic mice.

**Figure 5.**
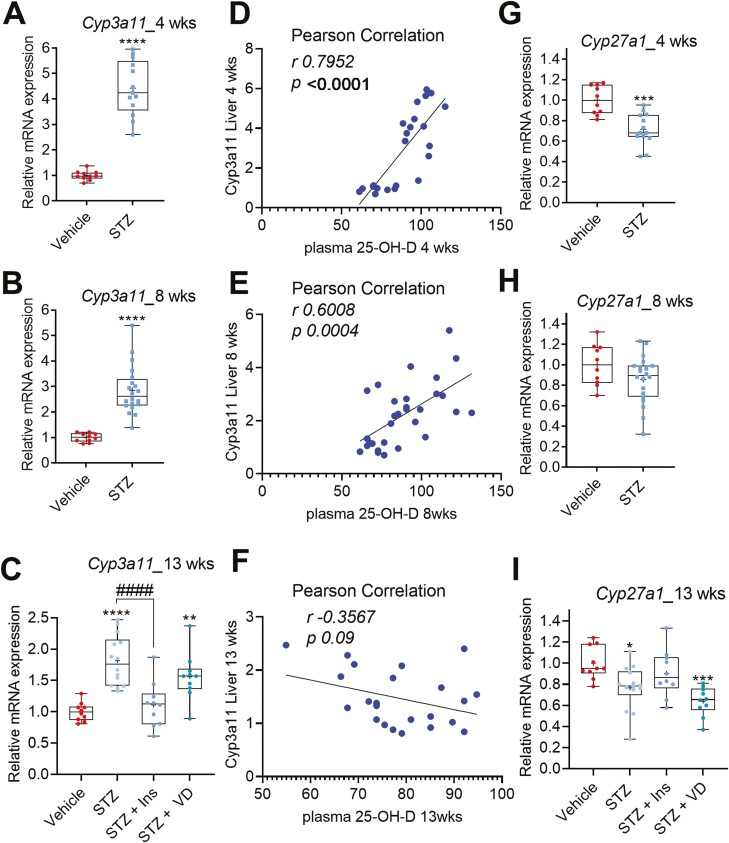
Effect of STZ-induced diabetes in mice on expression of *Cyp3a11* and *Cyp27a1* in liver. (A-C) The *Cyp3a11* was induced in the livers of diabetic mice after 4, 8, and 13 weeks. (D-F) The Pearson correlation between the liver *Cyp3a11* messenger RNA levels and the plasma 25-hydroxyvitamin D after 4, 8, and 13 weeks. (G-I) The effect of STZ-induced diabetes on the *Cyp27a1* expression in the liver after 4, 8, and 13 weeks. The box-and-whisker plots indicate the minimum, 25th percentile, median, 75th percentile, and maximum. In addition, the mean is indicated with +. *P or #*P* < 0.05, **P or ##*P* < 0.01, ***P or ###*P* < 0.001, and ****P or ####*P* < 0.0001. Abbreviations: Ins, insulin; STZ, streptozotocin; VD, vitamin D3.

*Cyp3a11* was significantly induced in the livers of STZ mice compared to nondiabetic controls in all the time points studied (Fig 5A-C). Moreover, there was a significant positive correlation between the liver *Cyp3a11* mRNA level and the plasma 25-OH-D level after 4 and 8 weeks (Fig 5D and 5E), while there was no correlation after 13 weeks (Fig 5F). In contrast, *Cyp27a1* was repressed in the livers of STZ mice after 4 and 13 weeks (Fig 5G, I), but not after 8 weeks (Fig 5H). Altogether, these data suggest that induction of other CYPs (eg, *Cyp3a11*), in the livers of STZ mice may compensate for the *Cyp2r1* repression and contribute to the higher plasma 25-OH-D detected in these mice.

### STZ-induced Diabetes Represses *Vdbp* Expression in the Liver

**Figure 6.**
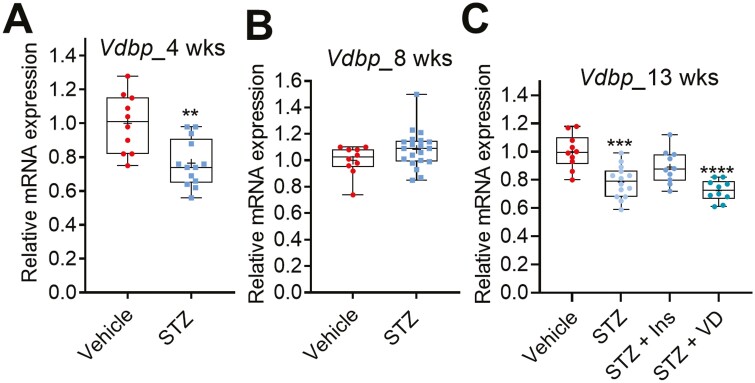
STZ-induced diabetes in mice represses *Vdbp* in the liver. (A-C) *Vdbp* messenger RNA levels in the livers of diabetic and nondiabetic control mice after 4, 8, and 13 weeks after STZ treatment. The box-and-whisker plots indicate the minimum, 25th percentile, median, 75th percentile, and maximum. In addition, the mean is indicated with +. *P < 0.05, **P < 0.01, ***P < 0.001, and **** *P* < 0.0001. Abbreviations: Ins, insulin; STZ, streptozotocin; VD, vitamin D3.

Plasma 25-OH-D binds with vitamin D binding protein (VDBP), and thus alterations in VDBP levels could affect the free plasma 25-OH-D concentration. We therefore investigated the possibility that STZ-induced diabetes could affect *Vdbp* regulation. *Vdbp* (also called *Gc*) mRNA was measured in the livers of nondiabetic and diabetic mice. Interestingly, the *Vdbp* mRNA level was significantly decreased after 4 and 13 weeks in the STZ mice compared to the nondiabetic controls (Fig 6A and 6C), while it was not affected after 8 weeks (Fig 6B). Treatment of the STZ mice with insulin attenuated the diabetes-mediated repression of *Vdbp* expression (Fig 6C), which may indicate a role for the insulin signaling in the regulation of *Vdbp* expression.

### STZ-induced Diabetes Represses the Expression of *Cyp2r1* in the Mouse Kidney

The liver is the major site expressing *Cyp2r1*; however, our previous studies have shown that *Cyp2r1* is expressed also in extrahepatic tissues (11). Therefore, we analyzed *Cyp2r1* expression in some extrahepatic tissues [ie, kidney, brown adipose tissue (BAT), and testis] of the nondiabetic and diabetic mice from the 13-week experiment.

**Figure 7.**
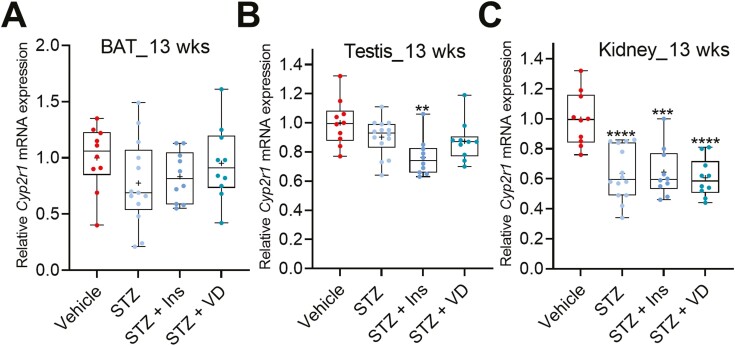
STZ-induced diabetes in mice represses *Cyp2r1* in the kidney. STZ-induced diabetes had no effect on the *Cyp2r1* expression in (A) the brown adipose tissue and (B) the testis, while (C) STZ-induced diabetes represses the *Cyp2r1* in the kidney compared to nondiabetic controls (13-week experiment). The box-and-whisker plots indicate the minimum, 25th percentile, median, 75th percentile, and maximum. In addition, the mean is indicated with +. *P < 0.05, **P < 0.01, ***P < 0.001, and ****P < 0.0001. Abbreviations: Ins, insulin; STZ, streptozotocin; VD, vitamin D3.

STZ-induced diabetes had no effect on the *Cyp2r1* expression in the BAT and testis (Fig 7A and 7B), while *Cyp2r1* was significantly repressed by 36% in the kidney of the STZ mice compared to the nondiabetic controls (Fig 7C). Similar to the liver, treatment of the STZ mice with insulin or vitamin D did not affect the repressive effect of the STZ-induced diabetes on the *Cyp2r1* expression in the kidney (Fig 7C).

### STZ-induced Diabetes Modulates the Expression of *Cyp27b1 and Cyp24a1* in the Kidney

**Figure 8.**
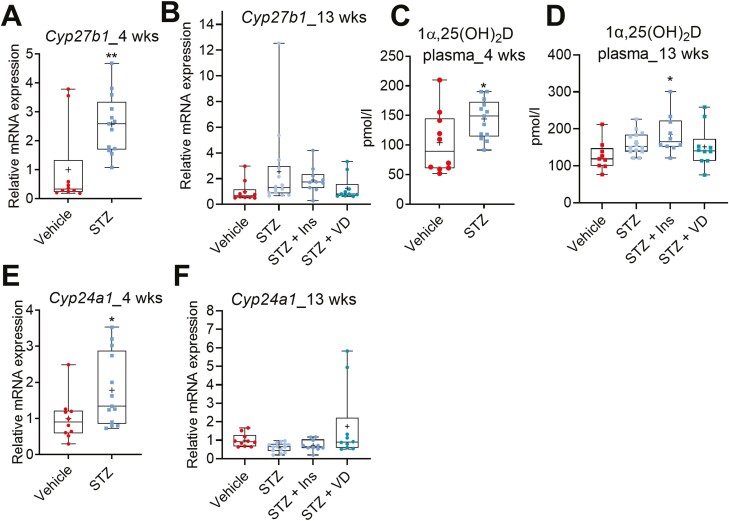
Effect of STZ-induced diabetes in mice on the expression of *Cyp27b1* and *Cyp24a1* in the kidney and plasma 1α25(OH)_2_D level. Effect of STZ-induced diabetes on *Cyp27b1* (A) after 4 weeks, and (B) after 13 weeks. Effect of STZ-induced diabetes on plasma 1α25(OH)_2_D level (C) after 4 weeks, and (D) after 13 weeks. Effect of STZ-induced diabetes on *Cyp24a1* (E) after 4 weeks and (F) after 13 weeks. The box-and-whisker plots indicate the minimum, 25th percentile, median, 75th percentile, and maximum. In addition, the mean is indicated with +. *P < 0.05 and **P < 0.01. Abbreviations: Ins, insulin; STZ, streptozotocin; VD, vitamin D3.

CYP27B1 is the sole vitamin D 1α-hydroxylase, responsible for conversion of 25-OH-D to 1α,25(OH)_2_D. The primary site of CYP27B1 expression is the kidney. Four weeks of STZ-induced diabetes significantly induced *Cyp27b1* in the kidney compared to the nondiabetic mice (Fig 8A). In contrast, after 13 weeks, the diabetes no longer affected *Cyp27b1* expression in the kidney (Fig 8B). Consistent with the *Cyp27b1* mRNA results, the plasma 1α,25(OH)_2_D level was increased after 4 weeks (Fig 8C), while it was not affected after 13 weeks of diabetes (Fig 8D). Insulin treatment modestly but significantly increased the plasma 1α,25(OH)_2_D in the diabetic mice (Fig 8D). Vitamin D supplementation had no effect on the plasma 1α,25(OH)_2_D in the diabetic mice (Fig 8D).

We also studied whether STZ-induced diabetes affects *Cyp24a1*, the major vitamin D catabolic enzyme in the kidney. Similar to the *Cyp27b1*, *Cyp24a1* expression was increased after 4 weeks (Fig 8E), while the expression was normalized after 13 weeks (Fig 8F).

### STZ-induced Diabetes Modulates the VDR Expression

**Figure 9.**
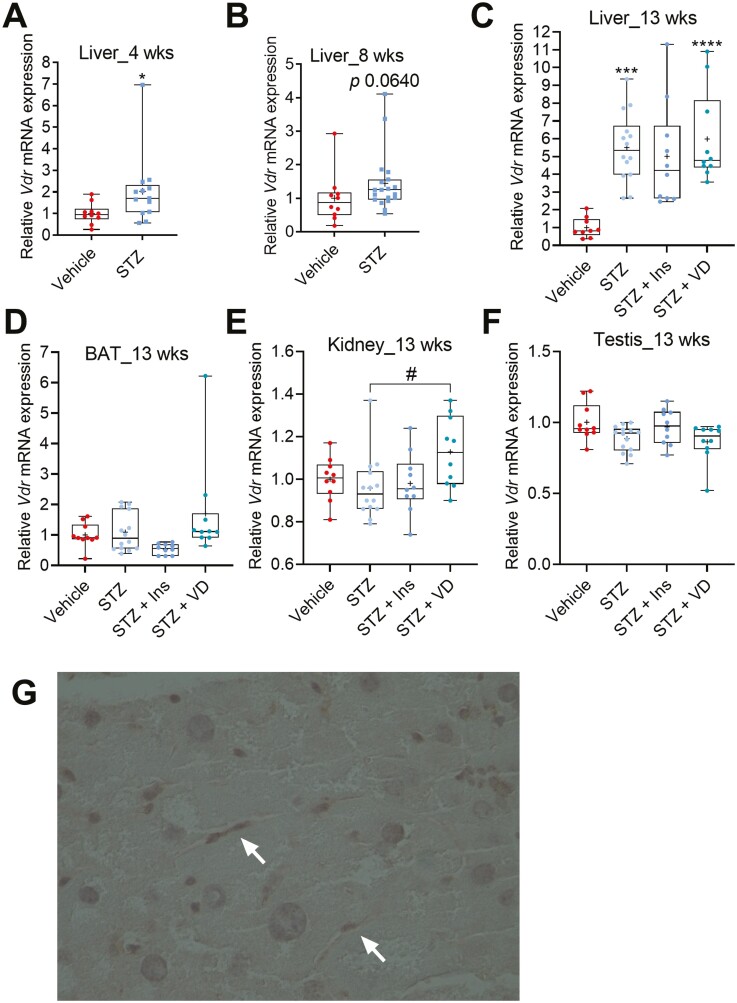
Effect of STZ-induced diabetes on the *Vdr* expression. (A and B) STZ-induced diabetes modestly induces *Vdr* in the livers of diabetic mice compared to nondiabetic mice after 4 and 8 weeks. (C) The *Vdr* expression was significantly higher in the liver of diabetic mice compared to nondiabetes mice after 13 weeks. (D-F) STZ-induced diabetes had no effect on the *Vdr* expression in the brown adipose tissue (BAT), kidney, and testis after 13 weeks. (G) Immunohistochemical detection of vitamin D receptor (VDR) in a STZ-treated mouse liver 13 weeks after treatment; 40-fold magnification. Examples of anti-VDR antibody-stained cells have been indicated with arrows. The box-and-whisker plots indicate the minimum, 25th percentile, median, 75th percentile, and maximum. In addition, the mean is indicated with +. *P or # *P* < 0.05, **P or ##*P* < 0.01, ***P or ###*P* < 0.001, and ****P or ####*P* < 0.0001. Abbreviations: Ins, insulin; STZ, streptozotocin; VD, vitamin D3.

Most of the biological functions of vitamin D are mediated through VDR (17). VDR is expressed widely in most tissues; however, liver expression is very low. To further analyze the effect of STZ-induced diabetes on the vitamin D biology, we analyzed the effect of STZ-induced diabetes on *Vdr* tissue expression. *Vdr* mRNA was detected in the livers of both the diabetic and control mice. However, the basal *Vdr* mRNA level in the liver of the nondiabetic mice was very low (based on a high threshold cycle value needed for detection). After 4 and 8 weeks of diabetes, there was a minor tendency for higher *Vdr* expression in the livers of the diabetic mice compared to the nondiabetic mice (Fig 9A and 9B). However, after 13 weeks, *Vdr* mRNA level was about 5-fold higher in the livers of the diabetic mice compared to the nondiabetic mice (Fig 9C). Under normal physiological conditions, *Vdr* is not expressed in the hepatic parenchymal cells; however, it is abundant in immune cells such as Kupffer cells (also known as liver macrophages) (18). Immunostaining of the livers with anti-VDR antibody indicated staining of the cells within the lumen of the liver sinusoids, a localization similar to the Kupffer cells. No positive staining was detected in the parenchymal hepatocytes (Fig. 9G).

*Vdr* was abundant in the extrahepatic tissues studied (ie, kidney, BAT and testis), but the expression was not affected by the STZ-induced diabetes (Fig 9D-9F).

## Discussion

Vitamin D deficiency has frequently been reported in type 1 diabetic patients; however, the molecular mechanisms are not known, and the causal relationship between low plasma 25-OH-D and development of type 1 diabetes is yet unclear (19). We recently reported that fasting, as well as obesity-induced diabetes, in mice repress *Cyp2r1*, the major vitamin D 25-hydroxylase, in the liver through PGC-1α/ERRα and GR pathways (10, 11). In the current study, we specifically studied how STZ-induced, insulin-deficiency diabetes with or without insulin therapy impacts *Cyp2r1* expression, vitamin D metabolism, and plasma 25-OH-D levels.

We observed that the STZ-induced diabetes represses *Cyp2r1* expression in the mouse liver. Insulin replacement therapy for 4 weeks in the STZ mice normalized the blood glucose and most of the diabetes-induced changes in the gene expression including *Pepck*, *Pgc-1α*, and *Cyp3a11*, among others. Curiously, insulin therapy did not rescue the diabetes-induced repression of *Cyp2r1* in the liver. A similar phenomenon was also observed in the kidney. The reasons for the lack of insulin sensitivity of the *Cyp2r1* regulation are currently unclear. However, it is known that peripheral delivery of insulin, bypassing the normal hepatic extraction, results in abnormal insulin balance and promotes insulin resistance (20). Furthermore, our treatment protocol included only treatment with long-acting insulin, and therefore the animals lacked the physiological insulin response to feeding. Nevertheless, the used insulin treatment normalized, either fully or partly, expression of the other diabetes-modified genes studied, indicating that the regulation of *Cyp2r1* is rather unusual.

PGC-1α and GR pathways are both known to participate in the *Cyp2r1* regulation (10). However, different from *Cyp2r1*, the diabetes-induced regulation of *Pgc-1α* and that of a GR target gene *Tat* was normalized by insulin treatment. Furthermore, no difference in the plasma corticosterone level was observed between the groups at the time of sacrifice. Therefore, the data suggest that neither PGC-1α nor GR plays any major role in the STZ-induced diabetes-mediated *Cyp2r1* repression.

In contrast to a previous report (21), STZ-induced diabetes did not decrease the plasma 25-OH-D level, and, in fact, the plasma 25-OH-D was higher after 4 and 8 weeks. It is noteworthy that the previous study was conducted in rats and not in mice, so the response might be species specific. However, in another rat study, liver microsomal conversion of vitamin D3 into 25-OH-D3 did not differ between the nondiabetic and the STZ rats (22). In our current study, the liver microsomal vitamin D 25-hydroxylase activity was increased in the livers of STZ-mice.

CYP2R1 is a major, but not the exclusive, vitamin D 25-hydroxylase in the liver (2), and other CYPs are considered to be potential vitamin D 25-hydroxylase enzymes (16). Thus, the lower CYP2R1 level could be compensated by induction of other vitamin D 25-hydroxylases. Currently it is unclear which enzymes, in addition to CYP2R1, would be the major vitamin D 25-hydroxylases in the mouse liver. *Cyp27a1* knockout in mice did not affect the plasma 25-OH-D, which indicate that the CYP27A1 is not a vitamin D 25-hydroxylase (16). CYP3A4 is highly expressed in the human liver and intestine (23). It has been demonstrated that CYP3A4 has 24-/25-hydroxylase activities toward vitamin D2, 1αOHD2, and 1αOHD3 (24). However, its significance in the vitamin D 25-hydroxylation is not clear. In our model, we demonstrated that the *Cyp3a11* (the mouse orthologue of human CYP3A4) was induced in the livers of the STZ mice. This agrees with a previous study showing that *Cyp3a11* is induced through constitutive androstane receptor–mediated mechanism in mouse models of type 1 diabetes (25). However, it is not known if CYP3A11 has any vitamin D 25-hydroxylase activity. Recently, Zhu et al demonstrated that vitamin D3 treatment increases the levels of hepatic *Cyp3a11*, and it was accompanied by high circulating plasma 25-OH-D and 1α,25(OH)_2_D levels (26). However, they did not measure the hepatic *Cyp2r1* expression level (26), and the contribution of CYP3A11 to the increased 25-OH-D is unclear. Interestingly, in our study, the hepatic *Cyp3a11* level was positively and significantly correlated with the plasma 25-OH-D level. Further studies are needed to directly investigate whether CYP3A11 could act as a vitamin D 25-hydroxylase in mouse liver. As we did not investigate expression of any other CYP enzymes, it also remains possible that other CYP enzymes with possible vitamin D 25-hydroxylase activity could also have been induced.

In our previous studies in the mouse model of HFD-induced obesity and type 2 diabetes, the plasma 25-OH-D decreased consistently with the lower hepatic *Cyp2r1* level (10, 11). Thus, the effect of *Cyp2r1* repression on 25-OH-D level appears to be different in type 1 and type 2 diabetes mouse models. In conjunction with other studies (27), we have previously performed RNA-sequencing of the livers from the HFD-fed obese mice shown to display both repressed *Cyp2r1* and low plasma 25-OH-D (the complete data sets are available at the NCBI’s Gene Expression Omnibus database accession no. GSE136667). Interestingly, in these obese and type 2 diabetic mice, there was a rather widespread repression of many hepatic CYP forms including *Cyp3a11* and other members on the Cyp3a subfamily. Thus, although the models of type 1 and type 2 diabetes have similar effects of the *Cyp2r1* expression, regulation of other CYP enzymes appear to be different, which may explain the observed difference in the vitamin D 25-hydroxylase activity.

Vitamin D is a lipophilic hormone, and adipose tissue is the major storage place. In uncontrolled diabetes, the metabolism shifts toward lipolysis in adipocytes (28). As a result, large quantities of lipids are released from the adipose tissue leading to loss of the subcutaneous and abdominal fats and subsequent release of vitamin D stored in the adipose tissue to the circulation. This process may supply large amount of vitamin D into the circulation and may be one important factor contributing to the increased plasma levels of 25-OH-D in the STZ mice after 4 and 8 weeks. After 13 weeks, there was already extensive loss of the adipose tissue in the untreated diabetic mice, and therefore no extra vitamin D could be released into the circulation. This might explain why STZ-induced diabetes appeared no longer to affect the plasma 25-OH-D level after 13 weeks.

VDBP is another key determinant of the plasma 25-OH-D levels (29). Reducing the hepatic VDBP production may result in lower plasma 25-OH-D (29). Similar to *Cyp2r1*, the *Vdbp* mRNA level was lower in the livers of STZ mice. However, unlike *Cyp2r1*, *Vdbp* suppression was corrected by insulin treatment, suggesting involvement of different mechanisms. In agreement with and supporting our results, an old study demonstrated that the plasma VDBP level decreases in the STZ-treated diabetic rats, and this was corrected by insulin treatment (30). A similar finding was also confirmed in humans with type 1 diabetes (31). Unfortunately, we were not able to measure plasma VDBP levels in this study.

The STZ-induced diabetes increased the *Cyp27b1* expression in the kidney after 4 weeks, while there was no longer an effect after 13 weeks. Plasma 1α,25(OH)_2_D, the metabolic product of CYP27B1, level followed the same pattern. This is in contrast with a previous study reporting that the plasma 1α,25(OH)_2_D decreases in the diabetic mice by 60% (32). On the other hand, similar to an earlier short-term study in rat, we observed that 4 weeks of STZ-induced diabetes increases the expression of the vitamin D catabolic enzyme *Cyp24a1* in the kidney (33). After 13 weeks, the expression was normalized. The increase in *Cyp24a1* expression after 4 weeks can probably be explained by the observed changes in the level of 1α,25(OH)_2_D, which is a major regulator of the *Cyp24a1* gene.

In summary, we demonstrated that *Cyp2r1* is repressed by the STZ-induced diabetes in the liver, and the effect could not be corrected by insulin therapy. While the *Cyp2r1* expression was repressed in the liver, the diabetic mice still retained their ability to 25-hydroxylate vitamin D and to produce sufficient level of plasma 25-OH-D. We propose that the lower hepatic *Cyp2r1* expression was compensated by increased expression of other CYP enzymes capable of catalyzing vitamin D 25-hydroxylation. Identification of these enzymes require further studies in the future.

## Data Availability

Original data generated and analyzed during this study are included in this published article or in the data repositories listed in the references.
